# Prognostic value and prevalence of complete right bundle branch block in an elderly population: a community-based 10-year prospective study

**DOI:** 10.18632/aging.103702

**Published:** 2020-10-06

**Authors:** Sherri Shih-Fan Yeh, Ching-Yu Julius Chen, I-Chien Wu, Chih-Cheng Hsu, Tzu-Yu Chen, Wei-Ting Tseng, Feng-Cheng Tang, Chi-Chung Wang, Chung-Chou Juan, Hou-Chang Chiu, Huey-Ming Lo, Dun-Hui Yang, Jyh-Ming Jimmy Juang, Chao Agnes Hsiung

**Affiliations:** 1 Department of Environmental and Occupational Medicine, National Taiwan University Hospital Hsin-Chu Branch, Hsin-Chu, Taiwan; 2 Cardiovascular Center and Division of Cardiology, Department of Internal Medicine, National Taiwan University Hospital and National Taiwan University College of Medicine, Taipei, Taiwan; 3 Institute of Population Health Sciences, National Health Research Institutes, Zhunan, Taiwan; 4 Department of Occupational Medicine, Changhua Christian Hospital, Changhua, Taiwan; 5 Department of Family Medicine, Mennonite Christian Hospital, Hualien, Taiwan; 6 Department of Surgery, Yuan’s General Hospital, Kaohsiung, Taiwan; 7 Department of Neurology, Shin Kong Wu Ho Su Memorial Hospital, Taipei, Taiwan; 8 College of Medicine, Fu-Jen Catholic University, New Taipei, Taiwan; 9 Section of Cardiology, Department of Internal Medicine, Shin Kong Wu Ho-Su Memorial Hospital, Taipei, Taiwan; 10 School of Medicine, Fu-Jen Catholic University, New Taipei, Taiwan; 11 Department of Radiology, Tainan Municipal Hospital, Tainan, Taiwan

**Keywords:** right bundle branch block, electrocardiogram, elderly

## Abstract

Complete right bundle branch block (CRBBB) occurs in 0.2% to 1.3% of the general population, but its prognostic significance in the geriatric population is unknown. We prospectively investigated the prevalence and prognostic value of CRBBB in individuals aged ≥65 years in a community-based population in Taiwan. A total of 5,830 community-dwelling individuals were prospectively recruited from 7 regions across Taiwan starting in December 2008 through March 2013. Those aged ≥65 years were included in the analysis (N=3,383). All subjects underwent a home visit and standardized medical exams and were followed up annually until the end of April 2019; cause of death was documented by citizen death records. The mean age of the study cohort was 73.5±5.9 years (65-104), and 47.21% were men. Among these individuals, 171 (5.05%) had CRBBB; the prevalence was higher in men (7.08%) than in women (3.25%). Subjects with CRBBB were older than those without CRBBB (75.4±6.5 vs. 73.4±5.9), and the frequency of CRBBB increased with age. Survival analysis revealed that all-cause mortality and cardiac mortality were similar in individuals with and without CRBBB during a mean follow-up of 92.6±23.6 months. CRBBB is not associated with increased risk of mortality in the geriatric population.

## INTRODUCTION

The world’s population is aging: virtually every country in the world is experiencing growth in the number and proportion of older persons in its population. According to data from the United Nations’ World Population Prospects, 1 in 11 people (9%) was over age 65 in 2019, and this proportion will increase to 1 in 6 people (15%) by 2050. It is therefore to be expected that the prevalence of diseases associated with old age, such as cardiovascular disease, will also rise.

The electrocardiogram (ECG) is a globally used, essential, inexpensive, and noninvasive technique to detect electric abnormalities of the heart. Disruption of normal electrical activity in the cardiac conduction system and delayed depolarization of the right ventricle consequently result in complete right bundle branch block (CRBBB). CRBBB is characterized by a QRS duration ≥0.12 seconds, a secondary R wave (R’), ST-segment depression and T-wave inversion in leads V1 or V2, and a wide slurred S wave in leads I, V5, and V6 of a 12-lead ECG. The prevalence and incidence of CRBBB is known to increase with age [1, 2]. CRBBB occurs in 0.3% to 1.6% of the general population; it can be an incidental finding on an ECG or a manifestation of underlying heart and pulmonary diseases [2–5].

The clinical significance of CRBBB in general populations has been extensively investigated. The age of enrolled individuals in previous studies ranged widely from 18 to 93 [6]. Although some previous studies included elderly individuals, the mean age of all previous study cohorts was 45-54 years old, which is below the typical cutoff for elderly populations (i.e., 65) [2, 6–13]. In addition, previous studies reported inconsistent results regarding the association between CRBBB and clinical outcomes such as all-cause mortality and cardiovascular mortality. For example, the Copenhagen City Heart Study enrolled individuals from the general population and reported that RBBB increased the risk of all-cause mortality and cardiac death in long-term follow-up [8]. However, the Swedish National registry data using hospital records showed that in men with RBBB, there was no increased risk of myocardial infarction (MI), heart failure, cardiac death or all-cause mortality [13]. Furthermore, the prognostic value of CRBBB was not specifically investigated in populations older than 65 years.

The Healthy Aging Longitudinal Study in Taiwan (HALST) is a prospective long-term community-based study of more than 5,800 adults that began in December 2008. It was designed to thoroughly examine the determinants of late-life health in an Asian population in Taiwan with long-term follow-up. The aim of the present study was to investigate the prevalence of CRBBB in the general population ≥65 years of age (which we define here as “elderly”), and to establish the prognostic implications of incidentally found CRBBB.

## RESULTS

**Figure 1:**
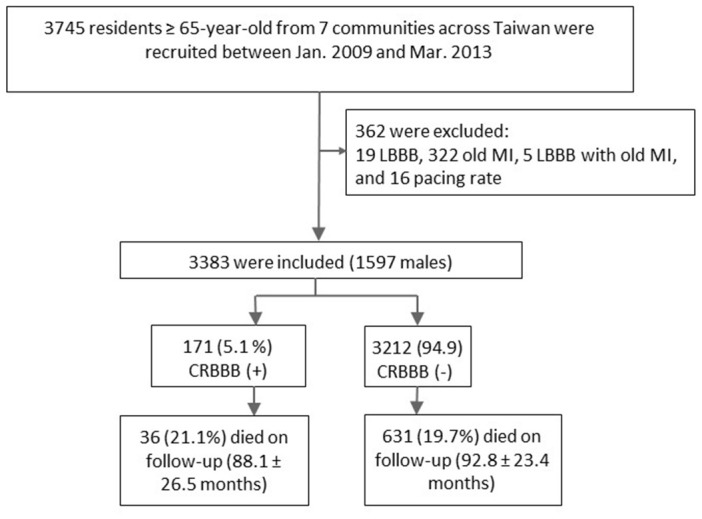
**Flowchart of study subject selection.**

Among 5,830 healthy people enrolled in the HALST cohort, 3,745 were aged ≥65; of these, 362 were excluded due to left bundle branch block, old MI, lung diseases, and pacing rate, leaving 3,383 individuals for analysis (Figure 1). Twenty-two of these individuals (0.65%) dropped out or were lost to follow-up during the study period. The mean age of the study cohort was 73.5±5.9 years, and 1,597 (47.21%) were men. Of the 3,383 individuals, 171 (5.05%) had CRBBB (Figure 1). Subjects with CRBBB were significantly older than those without CRBBB (75.4±6.5 vs. 73.4±5.9, *P*<0.0001; Table 1), and the prevalence of CRBBB was higher in men (n=113; 7.08%) than in women (n=58; 3.25%).

**Table 1:** General characteristics and prognosis of individuals with CRBBB and those without in the HALST cohort.

	**CRBBB(-) (n = 3212)**	**CRBBB(+) (n = 171)**	**P-value**
Men, n (%)	1484 (46.20)	113 (66.08)	<0.0001
Age, years	73.4±5.9	75.4±6.5	<0.0001
65-70, n (%)	1090 (33.94)	41 (23.98)	0.0011
70-74, n (%)	1010 (31.44)	48 (28.07)	
≥ 75, n (%)	1112 (34.62)	82 (47.95)	
Height, cm	157.8±8.4	160.4±8.7	<0.0001
Weight, kg	60.9±10.5	62.1±11.0	0.1447
Hypertension, n (%)	1595 (49.66)	87 (50.88)	0.7599
Diabetes mellitus, n (%)	658 (20.49)	37 (21.64)	0.7164
Dyslipidemia, n (%)	1014 (31.57)	51 (29.82)	0.6322
Stroke, n (%)	200 (6.23)	6 (3.51)	0.1476
ECG parameters			
Heart rate (beats/min)	67.5±10.3	67.0±10.8	0.5111
QRS, ms	91.8 (9.51)	143.1 (12.24)	<0.0001
QTc, ms	436.9±22.3	456.1±24.9	<0.0001
QRS axis, degree	25.6±35.5	18.3±51.2	0.0112
T axis, degree	40.9±31.8	21.6±31.0	<0.0001

Abbreviations: HALST, Healthy Aging Longitudinal Study in Taiwan; ECG, electrocardiogram; CRBBB(+), Complete right bundle branch block positive; CRBBB(-), Complete right bundle branch block negative.

**Figure 2:**
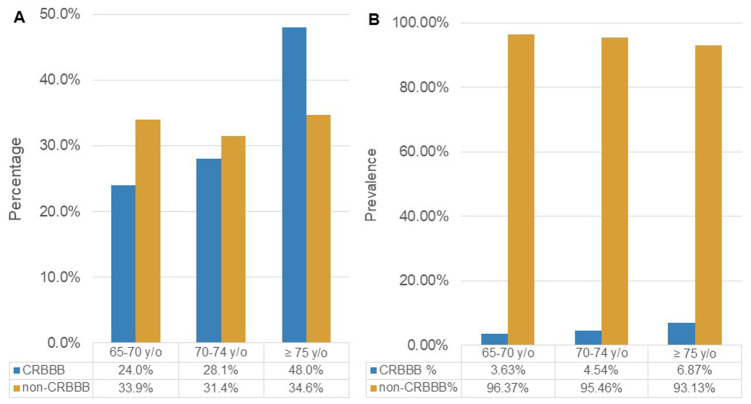
****(**A**) The age distribution among patients with and without CRBBB. (**B**) Prevalence of CRBBB and non-CRBBB in each age group. (Abbreviation: CRBBB, complete right bundle branch block).

**Figure 3:**
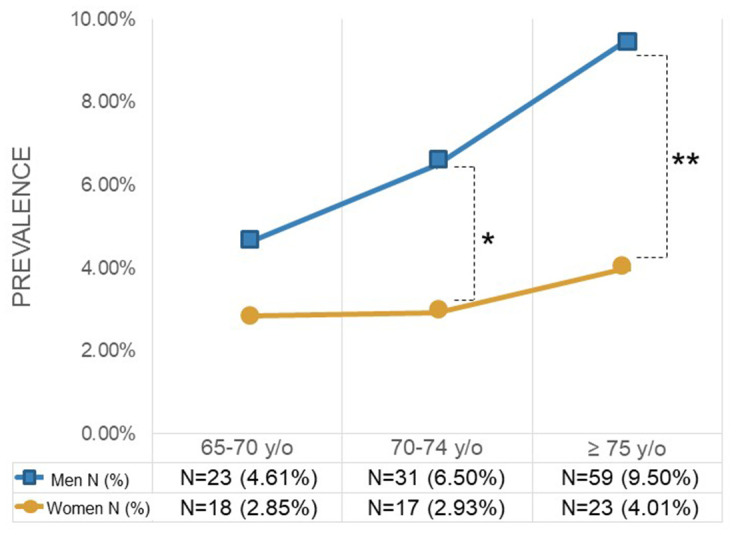
Prevalence of complete right bundle branch block by gender and age. *P<0.05, **P<0.005.

Figure 2A illustrates the age distribution among patients with and without CRBBB, showing that the group with CRBBB had a much higher percentage of people over 75. Figure 2B illustrates the prevalence of CRBBB in various age groups, showing that it significantly increased with age (*P*<0.001). On the other hand, the prevalence of CRBBB in elderly men was higher than that in elderly women regardless of age in this study (Figure 3). In additional subgroup analyses, we found that men older than 70 had significantly more CRBBB than women older than 70, whereas the difference was not significant below age 70 (*P*=0.0055 in 70-74 group, *P*=0.0002 in ≥75 group).

**Figure 4:**
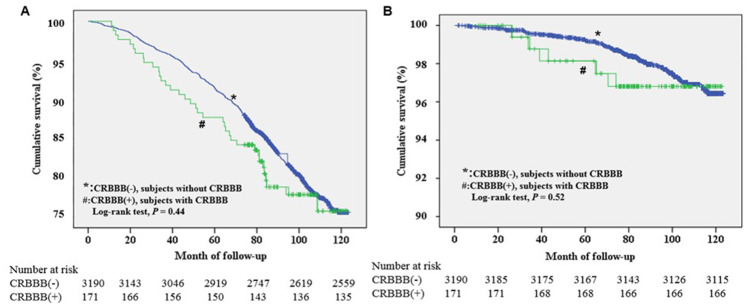
****Kaplan-Meier survival analysis of all-cause mortality (**A**) and cardiovascular mortality (**B**) in elderly individuals with and without complete right bundle branch block.

There were no significant differences in weight, body mass index, blood pressure, diabetes, dyslipidemia, previous events of stroke, atrial fibrillation, heart rate, and prognosis between those who had CRBBB and those who did not have CRBBB (Table 1). During an average follow-up of 92.6±23.6 months (7.7 years), neither all-cause mortality nor cardiovascular mortality were significantly different between the two groups (log-rank test, *P*=0.44, 0.52, respectively, Figure 4). After performing a Cox regression analysis controlling for potential confounders including stroke, hypertension, diabetes, dyslipidemia, and chronic kidney disease, CRBBB was still not significantly associated with all-cause mortality and cardiovascular mortality (P=0.48 and 0.56, respectively).

We also summarized the prevalence of CRBBB in community-based studies worldwide in Table 2. Because our study cohort is all elderly individuals (≥65 years), as expected, the average age of the individuals (73.5±5.9) in our study was the highest. In addition, the total prevalence of CRBBB (5.05%) in our study was the highest compared to the prevalence of other previously published studies (0.28-2.10%) and the mean worldwide prevalence (1.14%, an average of the values in Table 2). Regarding the gender difference, the prevalence of CRBBB in both men and women in our study (7.08% and 3.25%, respectively) was also the highest among the published community-based studies (0.95-2.10% and 0.42-1.25%, respectively).

**Table 2:** Summary of prevalence of CRBBB, age distribution, association with clinical outcomes of the published community-based studies worldwide

**Study**	**Year**	**Country**	**Screened population N**	**Gender**	**Age (years)**	**Subjects ≥ 65 years old**	**CRBBB (+)**			**All-cause mortality**	**Cardiovascular mortality**
							**N (%)**	**M (%)**	**F (%)**		
Van Der Ende et al.[6]	2017	Netherlands	149,803	Both	18-93 (mean: 45±13)	10,777	<65 y: 825 (0.59) ≥65 y: 369 (3.42)	817 (1.29)	377 (0.42)	NA	NA
Zhang et al.[7]	2015	USA	15,408	Both	45-64 (mean: 54)	0	228 (1.48)	-	-	NA	NA
Nakamura et al.[9]	2013	Japan	9,090	Both	≥30 (mean: 51)	?	117 (1.29) 64.2±12.4 y	76 (1.91)	41 (0.80)	Not increased	Not increased
Bussink et al.[8]	2013	Denmark	18,441	Both	≥20 (mean: 50±13)	1,015	166 (0.90) M: 64.0±12.3 y F: 61.6±10.2 y	119 (1.40)	47 (0.47)	increased	increased
Zhang et al.[10]	2012	USA	66,450	Only women	Mean: 63	?	832 (1.25) 65-67 y	-	832 (1.25)	Not increased	Not increased
Adesanya et al.[11]	2008	USA	52,582	Both	≥18	?	997 (1.90) 68.9 ±10 y	-	-	NA	NA
Aro et al.[12]	2010	Finland	10,899	Both	Mean: 44±9	0	31 (0.28)	-	-	Not increased	Not increased
Eriksson et al.[13]	2005	Sweden	7,392	Only men	Mean: 52±2	0	70 (0.95) 52.4±2.3 y	70 (0.95)	-	Not increased	Not increased
Taniguchi et. al.[18]	2003	Japan	2,722	Only men	Mean: 43.2±1.2	0	36 (1.32) 44.4±1.0 y	36 (1.32)	-	Not increased	Not increased
Thrainsdottir et al.[2]	1993	Iceland	18,762	Both	33-79	4677	193 (1.03)	126 (1.38)	67 (0.70)	Not increased	Not increased
Fleg et. al.[15]	1983	USA	1,142	Only men	?	?	24 (2.10) 75.4±6.5 y	24 (2.10)	-	Not increased	NA
Yeh et al. ^present study^	2020	Taiwan	3,383	Both	65-104 (mean 73.5±5.9)	3,383	171 (5.05) 75.4±6.5 y	113 (7.08)	58 (3.25)	Not increased	Not increased

Abbreviations: CRBBB, complete right bundle branch block; NA: not available, means no number was provided in the papers.

## DISCUSSION

The main findings of this study were as follows: 1) the prevalence of CRBBB was higher in men than in women and increased with age in both sexes; 2) the prevalence of CRBBB was much higher in this investigation compared with that in previous worldwide studies; 3) no significant association was shown between CRBBB and all-cause or cardiovascular mortality over an average 92.6±23.6 months of follow-up. To the best of our knowledge, this is the first study to examine the prevalence and prognosis of CRBBB in an elderly population with a mean age ≥65 years.

### Prevalence of CRBBB

Similar to previously published studies, our study showed that the prevalence of CRBBB was approximately twice to thrice as high in men compared with women [2, 6, 8, 9] and it was associated with increasing age [2, 3, 6, 8]. Van der Ende et al. reported that the prevalence of RBBB was as low as 0.58% before age 65 and increased to 3.4% at age 65 and older in The Lifelines Cohort Study in the Netherlands [6]. The Copenhagen City Heart Study found that the prevalence of CRBBB ranged from less than 1% below age 30 to more than 14% above age 80 in men [8]. The age range of our study participants was between 65 and 104, with a mean age of 73.5±5.9 years, and was the oldest of any community-based study published to date. Since the prevalence of ECG abnormalities was shown to increase with age [8], the CRBBB prevalence increased from 3.63% at ages below 70 years to 6.87% at ages of 75 and older in our study. Our study cohort also exhibited the highest overall CRBBB prevalence among community-based studies, supporting the theory of degeneration of the conduction pathways caused by aging [2].

### Clinical implications

CRBBB is considered a manifestation of a gradually evolving generalized process involving not only bundle branches but also structural changes in working myocardium [14]. However, this evolving change did not translate into a more pronounced risk of mortality in geriatric populations. Our finding may help clinicians in their assessments of geriatric individuals with incidental CRBBB and decrease unnecessary anxiety. On the other hand, CRBBB could mask the ECG phenotype of Brugada syndrome [15], which is a genetic disorder characterized by right bundle branch block and ST segment elevation in the precordial leads [16–20]. However, in elderly patients with CRBBB, the risk may be low even if the Brugada ECG is hidden by CRBBB [15].

### Long-term prognosis of elderly individuals with CRBBB

Previous community-based studies reported inconsistent results regarding the association between CRBBB and all-cause mortality and cardiovascular mortality. For example, Aro et al. evaluated the 12-lead ECGs of 10,899 middle-aged Finnish subjects from the general population (52% male; mean age 44±8.5 years) and reported that prolonged QRS duration (intraventricular conduction delay) is strongly associated with an increased risk of arrhythmic-related mortality [12]. Bussink et al. reported that CRBBB was associated with increased cardiovascular risk and all-cause mortality in 18,441 asymptomatic Danish individuals, whereas incomplete RBBB was not [8]. However, Fleg et al. reported that, in 1,142 men constituting the population of the Baltimore Longitudinal Study on Aging with a follow-up period averaging 8.4 years, RBBB was a manifestation of a primary abnormality of the cardiac conduction system but had no demonstrable adverse effect on long-term cardiac morbidity or mortality [21]. Eriksson et al. reported that in 7,392 men without a history of MI or stroke, there was no increased risk of cardiac or all-cause mortality in men with RBBB [13]. Some of these previous studies included only women or only men, and none of them were specific for individuals older than 65 years of age. To the best of our knowledge, ours is the first study to investigate an association between CRBBB and its prognostic value specifically for individuals older than 65 years of age on the basis of a 10-year follow-up study. In this study, we found that CRBBB was not associated with increased risk of all-cause mortality or cardiovascular mortality in asymptomatic individuals older than 65 years in the general population. Our findings provide an important reference for clinicians who are taking care of geriatric individuals.

### Limitations of the study

There are some limitations in this study. First, the population studied here was restricted to those with Han-Chinese ethnicity. It may not be applied to other ethnicities. Second, we do not have detailed clinical information such as echocardiographic assessment or coronary angiography or medications. For screening purposes, however, such information will rarely be available and thus the study mimics the setting in which screening would normally take place. Third, the HALST study is a well-designed cohort study with long term follow-up; all data used in the analysis including ECG data were carefully ascertained and read at central core labs by 2 independent cardiologists. However, as in other observational studies, residual confounding remains a possibility despite adjusting for several potential confounders.

## CONCLUSIONS

The prevalence of CRBBB is higher in generally healthy individuals older than 65 years in the community compared to younger populations. However, CRBBB alone was not associated with an increased risk of all-cause or cardiovascular mortality in long-term follow-up.

## PATIENTS AND METHODS

### Recruitment process and study population

**Figure 5:**
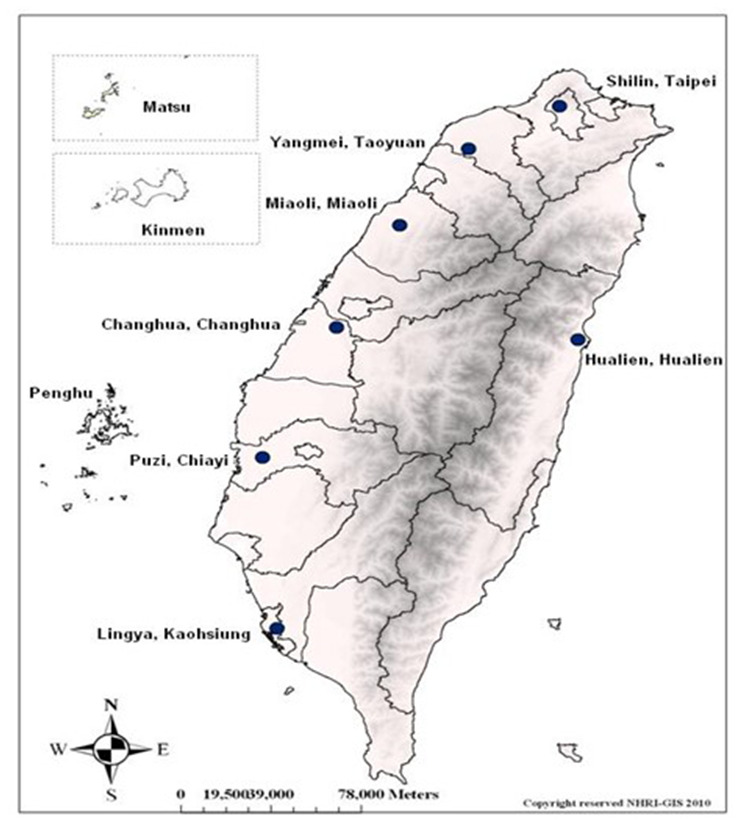
**Locations of the 7 participating sites across Taiwan in the HALST study.** Individuals were recruited from multiple regions across Taiwan, including 2 areas in the northern region, 2 in the central region, 2 in the southern region, and 1 in the eastern region. (Abbreviation: HALST, Healthy Aging Longitudinal Study in Taiwan).

The HALST cohort has been described in our previous work [22]. In brief, the recruitment process was as follows: adults living in the townships located within 2 kilometers of the study hospital in 7 regions across Taiwan were stratified by age, gender, and education level, and respondents were selected from each stratum by the systematic random sampling method. These 7 locations (Figure 5) cover both urban and rural areas, as well as different ethnic groups speaking different dialects, reasonably representing diverse sociodemographic characteristics of the Taiwanese background population. The eligibility criterion was age 65 years or older, and exclusion criteria were a highly contagious infectious disease or severe illness including malignancy undergoing active treatment, inability to ambulate, and institutionalization or hospitalization. Subjects with severe hearing, speech, mental, or cognitive impairments were also excluded because of their inability to answer questions accurately.

All of the study participants underwent home-visit assessments that included interviewer-administered questionnaires as well as standardized clinical and laboratory examinations in a hospital. The demographic variables explored include age, educational level, marital status, smoking status, alcohol intake, physical activity, and medications including antiarrhythmic drugs. Height, body weight, and blood pressure were recorded. Blood tests included lipid profile (total cholesterol, triglycerides, and HDL cholesterol), fasting glucose, and glycated hemoglobin (HbA1c). Hypertension was defined by self-report, the current use of anti-hypertensive medication, systolic blood pressure ≥130 mm Hg, or diastolic blood pressure ≥85 mm Hg at home. Dyslipidemia was defined by self-report, medication use, or high total cholesterol (≥240 mg/dL) or triglycerides (≥150 mg/dL) or reduced HDL cholesterol (men <40 mg/dL; women <50 mg/dL). Diabetes mellitus was defined by self-report, medication use, or high fasting plasma glucose (≥126 mg/dL) or HbA1c (≥6.5%).

This long-term prospective study was reviewed and approved by the Institutional Review Board of the National Health Research Institutes in Taiwan and their participating hospitals, and all participants provided written informed consent. Recruitment began in December 2008 and ended in March 2013. All enrollees were followed until death, and the cut-off for data collection in the current study was the end of April 2019. Among the 5,830 individuals enrolled in the HALST, we included 3,383 individuals ≥65 years of age who were free of documented heart disease at the time of enrollment (Figure 1). The participants with left bundle branch block, old MI, heart failure, severe valvular heart diseases, and arrhythmia were excluded from this study.

### ECG analysis and diagnostic criteria

All study subjects received 3 serial ECG examinations on the same day at enrollment. These 12-lead ECGs were recorded at 1-minute intervals using the standard settings of 10 mm/mV and 25 mm/s; PR, QRS, and corrected QT interval were automatically computed by Bazett’s formula. CRBBB was classified according to the Minnesota Code criteria (7-2-1) by a QRS duration ≥120 ms in a majority of beats in any of leads I, II, III, aVL, or aVF, plus R’ > R in lead V1 or V2; or QRS mainly upright, with an R-peak duration ≥60 ms in V1 or V2; or S duration > R duration in all beats in lead I or II, as in previous studies [9, 10, 23]. All ECGs were analyzed and interpreted by two independent cardiologists who were blinded to the participants’ clinical data. Those with left bundle branch block, old MI, pacing rhythm, lung diseases, or poor image quality on ECG were excluded from this study (Figure 1).

### Follow-up and outcomes

An annual follow-up telephone interview was carried out for all study participants. Information on the cause of death, if applicable, was determined by linking the database of death records from the Taiwan Ministry of Health and Welfare. We used International Classification of Diseases version 10 (ICD-10) codes to identify the cause of death and defined cardiovascular death by the ICD codes I01-I02.0, I05-I09, I20-I25, I27, and I30-I52. Survival curves were plotted by the Kaplan–Meier method, and the log-rank test was used for the comparison of the difference in mortality between patients with and without CRBBB.

### Statistical and survival analysis

We used either Chi-square or Fisher’s exact tests to compare categorical variables and one-way ANOVA for continuous variables. Inter-observer agreement was determined by the overall proportion of agreement across all ECGs using the Kappa statistic. The log-rank test and a Cox regression model were used for the comparison of the difference in mortality between participants with and without CRBBB. A two-tailed *P* value <0.05 was considered significant.
